# What influences general practitioners’ use of exercise for patients with chronic knee pain? Results from a national survey

**DOI:** 10.1186/s12875-016-0570-4

**Published:** 2016-12-19

**Authors:** Elizabeth Cottrell, Edward Roddy, Trishna Rathod, Mark Porcheret, Nadine E. Foster

**Affiliations:** Arthritis Research UK Primary Care Centre, Research Institute for Primary Care & Health Sciences, Keele University, Keele, Staffordshire ST5 5BG UK

**Keywords:** General practitioner, Attitude, Belief, Behaviour, Exercise, Chronic knee pain, Knee osteoarthritis, Questionnaire survey

## Abstract

**Background:**

Exercise is a recommended ‘core’ treatment for chronic knee pain (CKP), however it appears to be underused by general practitioners (GPs). While behavioural theories suggest that attitudes and beliefs influence behaviours, no single theory reliably predicts GPs’ behaviours. A theoretical analysis framework, developed from sociocognitive theories, was used to underpin investigation of the key influences associated with GPs’ use of exercise for patients with CKP, to inform future interventions to optimise GPs’ use of exercise.

**Methods:**

A cross-sectional postal questionnaire survey investigated UK GPs’ reported use of exercise based on a patient case vignette. Factors influencing GPs’ exercise use (behaviour) were examined using attitude statements, free-text questions and multiple response option questions related to factors within the analysis framework. Unadjusted logistic regression analyses explored the associations between GPs’ attitudes/beliefs and behaviour.

**Results:**

From a total sample of 5000 GPs, 835 (17%) returned a questionnaire. Most respondents (*n* = 729, 87%) reported that they would use exercise. Factors significantly associated with exercise use (OR (95% CI)) included GPs’ beliefs about their role (belief that GPs should give information on type, duration and frequency of exercise (30.71 (5.02,188.01)), beliefs about consequences (agreement that knee problems are improved by local (3.23 (1.94,5.39)) and general exercise (2.63 (1.38,5.02))), moral norm (agreement that GPs should prescribe all patients local (3.08 (1.96,4.83)) and general exercise (2.63 (1.45,4.76))), and GP-related beliefs about capabilities (prior experience of insufficient expertise to give detailed exercise information (0.50 (0.33,0.76)). Whilst perceived time limitations were not associated with exercise use (1.00 (0.33,3.01)), GPs who disagreed that they experienced time limitations were more likely to suggest general (2.17 (1.04,4.55)), or demonstrate local (2.16 (1.06,4.42)), exercises.

**Conclusion:**

GPs’ attitudes and beliefs are associated with their use of exercise for patients with CKP, particularly beliefs about role, responsibilities and skills in initiating exercise, and about the efficacy of exercise. Although the low response risks response bias, these results can inform future interventions to optimise GPs’ behaviour. The role of GP uncertainty and influences on clinical decision-making need further exploration, thus an amended analysis framework is suggested, which should be tested in future research.

**Electronic supplementary material:**

The online version of this article (doi:10.1186/s12875-016-0570-4) contains supplementary material, which is available to authorized users.

## Background

Chronic knee pain (CKP) in older adults, defined in this work as being synonymous with clinical knee osteoarthritis (OA) [1], is a common presentation to general practitioners (GPs) [2, 3]. Exercise, including both general aerobic and local strengthening exercise [4], is recommended as ‘core’ treatment for CKP [1] and empirical research evidence now unequivocally demonstrates that exercise improves pain and functioning in affected patients [5]. Despite being the primary source of formal medical advice for affected patients [6–9], GPs’ use of exercise (‘exercise use’) for CKP appears to be suboptimal [10] and attitudes about exercise for CKP are variable [10]. In order to appropriately target behaviour change interventions (e.g. to optimise GPs’ exercise use), it is logical to first identify key influences (or determinants of practice [11]) on GPs’ clinical behaviours. Indeed, recent evidence suggests that tailored intervention strategies, or strategies that are targeted at key influences on behaviour, can be effective [11].

For decades, sociocognitive theories [12, 13], have acknowledged that an individual’s attitudes and beliefs can influence their behaviours. For example, a GP who believes exercise for CKP to be ineffective may not recommend this to their patients. However, most recent theories recognise the non-linear association between attitudes, beliefs and behaviours resulting from multiple potential influences on an individuals’ behaviour. This is complicated further when a second person (e.g. a patient) is involved in the behaviour, as is the case for GPs’ clinical behaviours (the ways GPs act in the clinical context). Treatment decisions are particularly complex clinical behaviours [12]. Thus models designed to predict GPs’ clinical behaviour must be sufficiently sophisticated to account for individual GP-, service- and patient-related factors. Although multiple behavioural theories exist, no single theory robustly predicts behavioural intentions (*‘the expressed motivation to perform some behaviour or achieve some goal*’ [14]) or actual clinical behaviours among GPs. Given that the predictive ability of existing theoretical models is reduced among doctors when compared with nurses and other healthcare professionals (HCPs) [12], and that GPs have significantly different attitudes about clinical guidelines when compared with other doctors [15], a specific focus on GPs, rather than HCPs in general, is appropriate. Studies included in a previous systematic review examining GPs’ attitudes, beliefs and behaviours regarding exercise for CKP described GPs’ behaviours but did not explore factors influencing their behaviour [10]. Indeed, none of these studies explicitly referenced behavioural theory, although some alluded to this, for example acknowledging that clinical behaviours can be influenced by GP factors such as beliefs and ‘*cognitive rationales*’ [16–19], patient- [18] and guideline-related factors [20]. One more recent relevant study has explicitly used behavioural theory, the theory of planned behaviour (TPB), to investigate GP and patient beliefs about barriers to using conservative treatments for knee OA [21]. The aim of the current study was to investigate the key influences associated with GPs’ reported exercise use for patients with CKP using a cross-sectional questionnaire survey informed by an analysis framework developed using sociocognitive theories. The theoretical approach will first be described, before the study methods, themselves, are reported.

## Methods

### The analysis framework

**Fig. 1:**
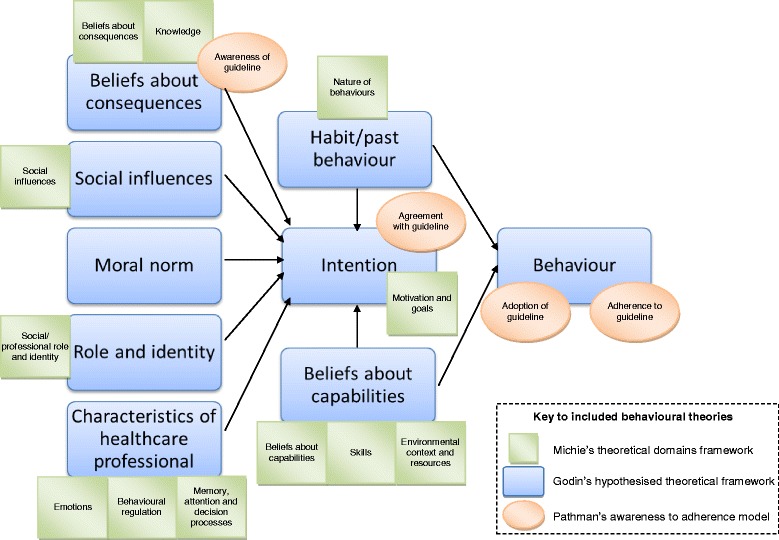
Analysis framework

Potential influences on GPs’ clinical behaviours can be identified and explained using the TPB, a long-established behavioural theory [13]. The TPB hypothesises that one’s attitudes, subjective norms and perceived behavioural control impact on one’s behavioural intentions and/or subsequent behaviour [13]. The inclusion of perceived behavioural control is particularly relevant to GPs’ clinical behaviours as it recognises that some behaviours are not under the complete control of the person performing them [13]; for example, key influences arise implicitly from working with patients and within service constraints. When tested among GPs [22–34], the TPB appears to be insufficient to comprehensively explain the behaviours of GPs in a variety of contexts [33]. No subsequent single theory has addressed this problem. Therefore an analysis framework was constructed for use in this study to explain the potential factors associated with GPs’ clinical behaviours, in this case, their exercise use for patients with CKP. Briefly, this was developed using three existing sociocognitive models; Michie’s theoretical domains framework (TDF) for predicting behaviour change (which identified 12 priority domains from a six-stage consensus exercise which included constructs from 33 relevant theories) [35], Godin’s hypothesised theoretical framework (which incorporates the elements of the TPB and other sociocognitive theories which best predict behaviours and behavioural intentions of HCPs) [12] and Pathman’s awareness-to-adherence model [36]. These three models were pragmatically combined by overlaying elements of the TDF and the awareness-to-adherence model onto Godin’s hypothesised theoretical framework. This prompted more comprehensive consideration of inherent factors that influence GPs’ behaviour, in addition to relevant organisational factors, such as the systems they work within and the people they work with (including patients), than using Godin’s or Pathman’s models alone. Further, it better acknowledges the relationships between all the factors, and that not all factors may influence behaviour at all times, which is not explicit in the TDF. The analysis framework, with all the elements of all included models, is shown in Fig. 1.

### Sample and sample size

The survey was mailed to 5000 UK GPs randomly selected from Binley’s database [37], which contains contact details of professionals working in UK general practices and is updated quarterly. A three-stage mailing process was used; at baseline (15^th^ January 2014) GPs were sent the questionnaire, at week two a reminder postcard was mailed to non-responders and at week four another copy of the questionnaire was sent to persistent non-responders. Questionnaires were sent to specific GPs with personalised cover letters, generated by the mailing database. A postage-paid return envelope, or postcard (for stage two), was enclosed. GPs were also given the opportunity to respond to an electronic version of the questionnaire if they preferred. Any recipients meeting exclusion criteria (not being a GP or not managing someone with CKP in the previous six months) were requested to indicate this and return the questionnaire uncompleted. Non-responders were invited to provide minimum data sets (MDS; gender, year of qualification, practice size and setting) using a reply slip attached to the cover letter (stages one and three) and using identical questions printed on the postage-paid return postcard (stage two). GPs’ completion and return of the questionnaire survey was taken as consent to participate in the study. No incentive was offered for completion of the questionnaire. Ethical approval for the study was obtained from Keele University Ethical Review Panel.

The sample size calculation was powered to investigate the association between GPs’ exercise use and their treatment orientation (determined using the adapted Pain Attitudes and Beliefs Scale for Physiotherapists (PABS_PT) tool, described further below). Without a validated method for interpreting differences in adapted PABS_PT subscale scores and anticipating greater differences in exercise use between GPs with more polarised biomedical and behavioural orientations, the adapted PABS_PT scores were split into quartiles and categorised as high (upper quartile) and low (lower quartile). Using a margin of error of 5% and a power of 90%, to detect an estimated difference in exercise use of 15% between those with scores in the upper versus lower quartiles on each subscale, a sample size of 748 was required [38]. After pragmatically rounding this up to 1000 and, based on the response of 20% obtained in the previous pilot study [39], a sample of 5000 UK GPs was required.

### Data collection

The analysis framework informed the development of the cross-sectional questionnaire survey tool used to collect data on GPs’ reported attitudes, beliefs and behaviours. Subsequent to refinement following a pilot survey with 172 UK GPs [39], the final eight-page survey tool (see Additional file 1) comprised five sections:

1. Demographics (‘*About you*’)
2. Vignette-based items investigating clinical behaviours, using a vignette depicting an initial GP presentation of a patient with CKP (‘*Clinical scenario of a patient with chronic knee pain*’)
3. Attitudes and beliefs about, and barriers towards, GPs managing CKP (‘*Chronic knee pain in general practice*’)
4. Attitudes and beliefs about CKP in general (‘*Your views about chronic knee pain*’)
5. Attitudes and beliefs about the role of exercise in managing CKP (‘*Your views about the role of exercise in treating chronic knee pain*’)

Additional file 2 illustrates how survey items mapped onto the analysis framework. Section 4 enquired about GPs’ attitudes and beliefs about CKP in general using an adapted version of the PABS_PT tool [40]. This tool, which was originally developed for use among physiotherapists in the context of low back pain (LBP) and subsequently adapted by Holden et al. [41] for use among physiotherapists for CKP, was further adapted for use among GPs in the context of CKP for this study. Responses from the 19-item tool are scored to provide two treatment orientation subscales [40, 42]. A high biomedical treatment orientation score suggests GPs interpret pain as being an indicator of (impending) physical damage, whereas a high behavioural subscale score suggests a more biopsychosocial approach, which shifts the focus away from underlying tissue damage and towards a more holistic view of the experience of pain, including psychosocial factors. This is relevant to enquiry of beliefs about consequences of exercise, as a high score on the biomedical subscale has previously been associated with HCPs viewing daily activities as harmful for LBP and providing advice to reduce work activities [42]. Attitude statements contained within Section 5 of the questionnaire were adapted from those developed by Holden et al. [43] who derived them from the MOVE consensus recommendations for HCPs to initiate exercise in the management patients with lower limb OA [4]. These attitude statements were relevant to the GPs’ beliefs about consequences, moral norm and role as they examined GPs’ attitudes and beliefs about the safety, efficacy and delivery of exercise.

### Data analyses

Prior to anonymisation of the mailing dataset, GPs’ practice postcodes were transformed into deprivation ranks, which were calculated separately for each country [44–47], and split into quintiles (from 1 = most deprived up to 5 = least deprived). When calculating the adapted PABS_PT subscale scores, missing data were dealt with in the following way [48]; when a maximum of one item was missing from a subscale, the missing item was imputed from the mean score of all the other items in that subscale, when more than one item from a subscale was missing, the whole subscale was classed as missing. Free-text responses, which primarily related to barriers to using exercise, underwent thematic analysis and unadjusted logistic regression analyses were undertaken to explore associations between factors within the analysis framework and the reported exercise use (used general or local exercise and/or referral to physiotherapist). Where attitudes and beliefs were established using Likert-scales, ambivalent or uncertain (neither disagree nor agree) responses were used as the reference category, with which (strongly) agree or (strongly) disagree responses were compared. Where associations of GP characteristics or previous experience of barriers were examined, the absence of the characteristic or barrier was used as the reference category. Where logistic regression analysis was not possible, Pearson Chi-square analysis or Fisher’s Exact Test were undertaken as appropriate. All analyses were performed using IBM SPSS Statistics (Version 20).

Time constraints were frequently raised during the study, among reasons for non-response and within survey responses, but no association with the overall exercise use was found in the *a priori* analyses. To assess whether time constraints affected how, rather than if, GPs used exercise, logistic regression was used in *a posteriori* analysis to explore the association between agreement with ‘*time constraints prevent GPs from providing advice on individual exercises for CKP*’ and general and local exercise delivery methods.

## Results

### Response

Of the 5000 UK GPs sent the questionnaire, 58 met the exclusion criteria and 835 (adjusted response 17%) returned a completed questionnaire. An additional 470 GPs (10%) responded with a MDS, of whom most (*n* = 408; 87%) cited insufficient time as the primary reason for not completing the full questionnaire. Full questionnaire responders had been qualified for a shorter time and were more likely to work in a less-deprived area than MDS responders. Gender, practice type and number of GPs in the practice did not differ between full questionnaire and MDS responders (Table 1). Many free-text responses were mapped to existing response headings or themes identified within the previous pilot study. However, there was a rich seam of novel themes arising among barriers to using exercise; these are presented in Table 2.

**Table 1:** Demographic details of full questionnaire respondents versus those providing minimum data sets

Variable	Category	Response type		Statistic (95% CI)
		MDS (*n* = 470)	Completed questionnaire (*n* = 835)	
Gender	Male	247 (53%)	401 (49%)	OR 1.00
	Female	219 (47%)	417 (51%)	OR 1.17 (0.93,1.47)
Practice area deprivation	Most deprived	121 (26%)	181 (22%)	**OR 0.63 (0.45,0.89)**
	Second most deprived	106 (23%)	156 (19%)	**OR 0.62 (0.44,0.88)**
	Mid-deprived	85 (18%)	202 (24%)	OR 1.00
	Second least deprived	84 (18%)	160 (19%)	OR 0.80 (0.56,1.16)
	Least deprived	73 (16%)	135 (16%)	OR 0.78 (0.53,1.14)
Practice type	Urban	254 (56%)	449 (54%)	OR 1.00
	Semi-rural	155 (34%)	275 (33%)	OR 1.00 (0.78,1.29)
	Rural	43 (10%)	103 (13%)	OR 1.36 (0.92,2.00)
Mean (SD) years since qualification		21.6 (10.0)	18.4 (10.3)	Mean difference = -**3.24 (−4.41,-2.07)**
Mean (SD) no of GPs in respondent’s practice		6.4 (3.7)	6.4 (3.2)	Mean difference = <0.01 (−0.38,0.39)
Information only requested in questionnaire				
Type of GP	GP partner	- - -	656 (79%)	
	Salaried GP	- - -	151 (18%)	
	Locum GP	- - -	20 (2%)	
	Other	- - -	5 (1%)	
GP with special interest in musculoskeletal conditions		- - -	50 (6%)	
Received postgraduate education which contained education about CKP		- - -	319 (39%)	
Personal experience of CKP		- - - -	166 (20%)	

Maximum missing data for any cell was 6%

*CI* confidence interval, *CKP* chronic knee pain, *GP* general practitioner, *MDS* minimum data set, *OR* odds ratio

Results reaching statistical significance are captured in bold

**Table 2:** Summary of themes, concepts and examples of free text responses regarding barriers to the use of exercise for CKP

Theme	Concepts	Given as a response option	Example of coded response
Service-related	Insufficient time in consultations	✓	[Nil additional free text comments given]
	Difficulty accessing physiotherapy	✓	“Takes 18 weeks to see a physio”
	Limitations to accessing services	✗	“Pressure on reducing referrals” “Loss of local fitness initiative” “Lack of any facilities in our local area for people to group exercise - no sports or leisure facility.” “Cost of exercise to patient e.g. Gym membership”
	My GP colleagues do not use or value exercise	✓	[Nil additional free text comments given]
	Services do not meet expectations	✗	“…some patients wait for 3/12 and once they’ve seen they’ve been given a leaflet to do exercise at home, this does not meet patients’ expectations” “Only get 2 physio sessions if we refer them” “Physiotherapy appointments are not long enough”
	Geographical problems	✗	“Remote location of practice deters patients from travelling to a gym” “Patients are too scared to walk in local area”
GP-related	Insufficient expertise to give detailed information	✓	[Nil additional free text comments given]
	Uncertainty about the most appropriate type of exercise	✓	[Nil additional free text comments given]
	Uncertainty about the effects of exercise	✓	[Nil additional free text comments given]
	Uncertainty about the safety of exercise	✓	[Nil additional free text comments given]
	Cannot access necessary resources	✗	“Lack of structured approach I know the info is out there somewhere - don’t have time or energy to search” “Detailed leaflet sounds good - if I have time I will look at arthritis UK website”
	GP does not prioritise exercise	✗	“Perhaps I should give it a higher priority”
	Unclear what physio offers	✗	“Little feedback from physiotherapy about advice offered/range of services”
Patient-related	Patients prefer other management options	✓	“When mention physiotherapy and exercise most patients don’t want this - ‘they just give you exercises and it makes the pain worse’”
	Exercise does not match patient needs/expectations	✗	“Patients want a ‘quick fix’ losing weight and increasing exercise is more difficult” “Patients so overweight that they cannot even consider exercise - in fact this annoys them” “Patient appearing so debilitated by chronic pain that exercise cannot be tolerated” “Patient refusal to engage with regular exercise due to perceived time constraints and fear of harming themselves” “I advise on quad strengthening, patients often sceptical this is enough to help relieve their symptoms” “Specialist colleagues appear to always want MRI/CT/xray/arthroscopy + people talk to each other (I had this + the specialist did….)”
	Achieving patient behaviour change is difficult	✗	“Very difficult to get many patients to change lifestyle sufficiently to effect enough real change to help knee pain” “Many pts are lazy!” “patient reluctance” “Requires significant patient re-education and elements of motivational interviewing so potentially v time consuming”
Other	Other	✓	“physiotherapy (referral) needs to be prioritised”

### Influences on the use of exercise

Most respondents (*n* = 729; 87%) reported that they would use exercise in some way to manage the vignette patient. The associations between GPs’ attitudes/beliefs and their exercise use were assessed according to the analysis framework (Fig. 1 and Additional file 2). Factors significantly associated with GPs’ exercise use were their beliefs about their role and about consequences of using exercise, their moral norm and GP-related beliefs about capabilities. These factors are first described, before those which were not found to be significantly associated with GPs’ exercise use (see Additional file 3 for a summary of results).

#### Beliefs about role

GPs’ beliefs about their role, specifically regarding the inclusion of exercise into their management of a patient with CKP, were significantly associated with their exercise use. The greater the GPs perceived their role to be in initiating exercise, the more likely they were to use exercise (Table 3). Exercise use was significantly increased among GPs agreeing that it is part of their job to reassure patients about the safety of exercise for CKP (OR 3.57 (95% Confidence Interval 1.91,6.59); Table 3), that they should educate patients with CKP about how to change their lifestyle for the better (OR 2.43 (1.22,4.82); Table 4), and that it is part of their job to provide patients with a written management plan (OR 2.21 (1.29,3.80); Table 3). Disagreement with these statements was not significantly associated with exercise use. Responses to other items relating to role which were not associated with GPs’ exercise use included, beliefs about it being the patients’ own responsibility to continue doing their exercise programme, beliefs that GPs should follow-up patients to monitor the extent of their continuation of exercises (Table 4) and beliefs about their role in managing CKP in general (Table 3).

**Table 3:** Use of exercise according to GPs’ beliefs about their role

Role		Use of exercise for vignette patient		OR (95% CI) for use of exercise
		No n (%)	Yes n (%)	
GPs’ beliefs about their role in managing people with CKP in general				
It is part of my job to manage people with CKP	Neither agree nor disagree	1 (10%)	9 (90%)	1.00
	(Strongly) disagree	0 (0%)	8 (100%)	**- - - -**
	(Strongly) agree	103 (13%)	710 (87%)	0.77 (0.10,6.11)
It is part of my job to provide patients with CKP with a written management plan	Neither agree nor disagree	53 (15%)	309 (85%)	1.00
	(Strongly) disagree	31 (16%)	159 (84%)	0.88 (0.54,1.43)
	(Strongly) agree	20 (7%)	258 (93%)	**2.21 (1.29,3.80)**
GPs’ beliefs about their role in including exercise				
It is part of my job to reassure patients about the safety of exercise for CKP	Neither agree nor disagree	17 (30%)	39 (70%)	1.00
	(Strongly) disagree	4 (33%)	8 (67%)	0.87 (0.23,3.29)
	(Strongly) agree	83 (11%)	679 (89%)	**3.57 (1.93,6.59)**
Which statement best describes your role in including exercise in the management plan of a patient with CKP?	I have no role in including exercise in the management plan	5 (42%)	7 (58%)	1.00
	I inform patients that exercise is a management option	35 (34%)	67 (66%)	1.37 (0.40,4.62)
	I advise patients to use exercise to manage their knee pain	37 (14%)	238 (87%)	**4.60 (1.39,15.24)**
	I recommend the types of exercise patients could use	24 (7%)	329 (93%)	**9.79 (2.89,33.17)**
	I give information on the type, frequency and duration of specific exercises	2 (2%)	86 (98%)	**30.71 (5.02,188.01)**

*CI* confidence interval, *CKP* chronic knee pain, *GP* general practitioner, *OR* odds ratio

CI not spanning 1.0 are captured in bold

**Table 4:** Use of exercise according to MOVE consensus-derived attitude statement responses: statements relating to the delivery of, and adherence to, exercise

Attitude statement	Response to attitude statement	Used exercise for the vignette patient		Odds ratio (95% CI) for use of exercise
		Yes	No	
Exercise for CKP is most beneficial when it is tailored to meet individual patient needs^a^	Neither disagree or agree	13 (18%)	60 (82%)	1.00
	(Strongly) disagree	0 (0%)	9 (100%)	- - - -
	(Strongly) agree	91 (12%)	650 (88%)	1.55 (0.82,2.93)
A standard set of exercises is sufficient for every patient with chronic knee problems^a^	Neither disagree or agree	38 (13%)	254 (87%)	1.00
	(Strongly) disagree	54 (13%)	367 (87%)	1.02 (0.65,1.59)
	(Strongly) agree	9 (8%)	99 (92%)	1.65 (0.77,3.53)
GPs should educate CKP patients about how to change their lifestyle for the better^b^	Neither disagree or agree	12 (24%)	38 (76%)	1.00
	(Strongly) disagree	3 (33%)	6 (67%)	0.63 (0.14,2.92)
	(Strongly) agree	88 (12%)	676 (89%)	**2.43 (1.22,4.82)**
It is important that people with CKP increase their overall activity levels^a^	Neither disagree or agree	17 (22%)	62 (79%)	1.00
	(Strongly) disagree	5 (50%)	5 (50%)	0.27 (0.07,1.06)
	(Strongly) agree	82 (11%)	653 (89%)	**2.18 (1.22,3.91)**
How well a patient complies with their exercise programme determines how effective it will be^a^	Neither disagree or agree	12 (13%)	79 (87%)	1.00
	(Strongly) disagree	7 (32%)	15 (68%)	**0.33 (0.11,0.96)**
	(Strongly) agree	85 (12%)	627 (88%)	1.12 (0.59,2.14)
GPs should follow-up patients to monitor extent of continuation of exercises^b^	Neither disagree or agree	37 (12%)	265 (88%)	1.00
	(Strongly) disagree	33 (13%)	212 (87%)	0.90 (0.54,1.48)
	(Strongly) agree	33 (12%)	243 (88%)	1.03 (0.62,1.70)
It is the patient’s own responsibility to continue doing their exercise programme^b^	Neither disagree or agree	6 (13%)	42 (88%)	1.00
	(Strongly) disagree	2 (29%)	5 (71%)	0.36 (0.06,2.27)
	(Strongly) agree	96 (12%)	675 (88%)	1.00 (0.42,2.43)

*CI* confidence interval, *CKP* chronic knee pain, *GP* general practitioner

^a^ = Beliefs about consequences; ^b^ = Role and identity

CI not spanning 1.0 are captured in bold

#### Beliefs about consequences: knowledge and attitudes about the efficacy of exercise

Prior experience of uncertainties about the effects of exercise was associated with significantly lower exercise use (22/43 (51%); OR 0.13 (0.07,0.24)) when compared with those who had not experienced this barrier (707/792 (89%)). Exercise use was significantly higher among those GPs who agreed that knee problems are improved by local (OR 3.23 (1.94,5.39)) or general (OR 2.63 (1.38,5.02)) exercises compared to those who neither agreed nor disagreed (Table 5). No significant associations with exercise use were identified among those disagreeing with these items. Beliefs about increasing strength of the muscles around the knee or overall activity levels stopping knee problems getting worse were not significantly associated with GPs’ exercise use (Table 5).

**Table 5:** Use of exercise according to MOVE consensus-derived attitude statement responses: statements relating to the benefits of exercise

Attitude statement	Response to attitude statement	Used exercise for the vignette patient		Odds ratio (95% CI) for use of exercise
		No	Yes	
GPs should prescribe quadriceps strengthening exercises to every patient with CKP^a^	Neither disagree or agree	42 (23%)	142 (77%)	1.00
	(Strongly) disagree	12 (18%)	56 (82%)	1.38 (0.68,2.81)
	(Strongly) agree	50 (9%)	520 (91%)	**3.08 (1.96,4.83)**
GPs should prescribe general exercise, for example, walking or swimming, for every patient with CKP^a^	Neither disagree or agree	17 (25%)	50 (75%)	1.00
	(Strongly) disagree	3 (13%)	21 (88%)	2.38 (0.63,8.99)
	(Strongly) agree	84 (11%)	649 (89%)	**2.63 (1.45,4.76)**
Knee problems are improved by quadriceps strengthening exercises^b^	Neither disagree or agree	26 (28%)	67 (72%)	1.00
	(Strongly) disagree	0 (0%)	3 (100%)	- - - -
	(Strongly) agree	78 (11%)	650 (89%)	**3.23 (1.94,5.39)**
Knee problems are improved by general exercise, for example, walking or swimming^b^	Neither disagree or agree	14 (26%)	40 (74%)	1.00
	(Strongly) disagree	0 (0%)	4 (100%)	- - - -
	(Strongly) agree	90 (12%)	676 (88%)	**2.63 (1.38,5.02)**
Quadriceps strengthening exercises for the knee are safe for everybody to do^b^	Neither disagree or agree	44 (18%)	200 (82%)	1.00
	(Strongly) disagree	15 (13%)	105 (88%)	1.54 (0.82,2.90)
	(Strongly) agree	45 (10%)	412 (90%)	**2.01 (1.29,3.15)**
General exercise, for example, walking or swimming, is safe for everybody to do^b^	Neither disagree or agree	26 (20%)	106 (80%)	1.00
	(Strongly) disagree	14 (13%)	91 (87%)	1.59 (0.79,3.24)
	(Strongly) agree	64 (11%)	519 (89%)	**1.99 (1.21,3.28)**
Exercise is effective for patients if an x-ray shows severe knee osteoarthritis^b^	Neither disagree or agree	42 (16%)	219 (84%)	1.00
	(Strongly) disagree	24 (18%)	108 (82%)	0.86 (0.50,1.50)
	(Strongly) agree	38 (9%)	391 (91%)	**1.97 (1.24,3.15)**
Exercise works just as well for everybody, regardless of the amount of pain they have^b^	Neither disagree or agree	32 (13%)	207 (87%)	1.00
	(Strongly) disagree	55 (14%)	349 (86%)	0.98 (0.61,1.57)
	(Strongly) agree	17 (9%)	163 (91%)	1.48 (0.80,2.76)
Increasing the strength of the muscles around the knee stops the knee problem getting worse^b^	Neither disagree or agree	37 (15%)	203 (85%)	1.00
	(Strongly) disagree	19 (15%)	109 (85%)	1.05 (0.57,1.91)
	(Strongly) agree	48 (11%)	408 (90%)	1.55 (0.98,2.46)
Increasing the overall activity levels stops the knee problem getting worse^b^	Neither disagree or agree	39 (13%)	270 (87%)	1.00
	(Strongly) disagree	28 (18%)	130 (82%)	0.67 (0.40,1.14)
	(Strongly) agree	37 (10%)	318 (90%)	1.24 (0.77,2.00)

*CI* confidence interval, *CKP* chronic knee pain, *GP* general practitioner

^a^ = Moral norm; ^b^ = Beliefs about consequences

CI not spanning 1.0 are captured in bold

#### Beliefs about consequences: awareness of management recommendations

A small but statistically significant difference in exercise use was observed among GPs who reported having read the National Institute for Health and Care Excellence (NICE) osteoarthritis guidelines [1] (291/321 (91%)) compared with those who did not (427/501 (85%); OR 1.68 (1.07,2.64)). Exercise use was significantly greater among GPs who disagreed that exercise should only be used after drug treatment has been tried (622/702 (89%); OR 2.10 (1.22,3.63)) compared to those who neither disagreed nor agreed (74/94 (79%)); agreement with this statement was not significantly associated with exercise use (24/28 (86%); OR 1.62 (0.50,5.22)). Exercise use was significantly higher among those who agreed that it is important that people with CKP increase their overall activity levels compared with those who neither agreed nor disagreed (OR 2.18 (1.22,3.91)). Disagreement with this item was not significantly associated with exercise use. Disagreement with the statement ‘*How well a patient complies with their exercise programme determines how effective it will be*’ was significantly associated with lower exercise use (OR 0.33 (0.11,0.96)). Beliefs about a standard set of exercises being sufficient for every patient with CKP were not associated with exercise use.

#### Beliefs about consequences: factors that may be perceived to influence efficacy of exercise

Exercise use was significantly greater among GPs who agreed with the statement ‘*exercise is effective for patients if an x-ray shows severe knee OA’* (OR 1.97 (1.24,3.15); however there was no association found among GPs who disagreed with this statement (Table 5). Although exercise use was greater among GPs who perceived the patient’s symptoms to be (very) mild (77/82, 94%) compared to those who perceived them to be (very) severe (92/106, 87%), this was not statistically significant (OR 2.34 (0.81,6.80)). Exercise use among GPs who thought the underlying knee damage was (very) severe (50/64 (78%)) was lower than among those who thought it was moderate (471/530 (89%); OR 2.24 (1.17,4.29), significant) or (very) mild (194/222 (87%); OR 1.94 (0.95,3.96), non-significant). However, exercise use was not associated with GPs’ using the term ‘*wear and tear*’ to describe the diagnosis (605/691 (88%) versus 122/139 (88%) who did not; OR 0.98 (0.56,1.71)), GPs’ requesting use of knee x-ray for the vignette patient (487/564 (86%) versus 242/271 (89%) who did not; OR 0.76 (0.48,1.19)), nor GPs’ beliefs about the patients’ future and causal factors for CKP (Table 6).

**Table 6:** Unadjusted logistic regression examining the association between the use of exercise and risk factors for CKP

Risk factor	Agreement with item being risk factor	Not using exercise (%)	Using exercise (%)	Odds ratio (95% CI) for use of exercise
Non-modifiable				
Hereditary/runs in the family	Neither agree or disagree	30 (12%)	212 (87%)	1.00
	(Strongly) disagree	25 (13%)	163 (87%)	0.92 (0.52,1.63)
	(Strongly) agree	47 (12%)	338 (88%)	1.02 (0.62,1.66)
Ageing	Neither agree or disagree	8 (13%)	53 (87%)	1.00
	(Strongly) disagree	1 (5%)	18 (95%)	2.72 (0.32,23.24)
	(Strongly) agree	95 (13%)	653 (87%)	1.04 (0.48,2.25)
Changes consistent with OA seen on x-ray	Neither agree or disagree	22 (10%)	207 (90%)	1.00
	(Strongly) disagree	9 (12%)	67 (88%)	0.79 (0.35,1.80)
	(Strongly) agree	73 (14%)	447 (86%)	0.65 (0.39,1.08)
Modifiable				
Accident or injury	Neither agree or disagree	7 (22%)	25 (78%)	1.00
	(Strongly) disagree	2 (22%)	7 (78%)	0.98 (0.17,5.82)
	(Strongly) agree	94 (12%)	693 (88%)	2.06 (0.87,4.90)
A person’s own mental attitude	Neither agree or disagree	23 (14%)	140 (86%)	1.00
	(Strongly) disagree	17 (22%)	61 (78%)	0.59 (0.29, 1.18)
	(Strongly) agree	64 (11%)	518 (89%)	1.33 (0.80,2.22)
A person’s emotional state	Neither agree or disagree	19 (14%)	115 (86%)	1.00
	(Strongly) disagree	12 (17%)	59 (83%)	0.81 (0.37,1.79)
	(Strongly) agree	73 (12%)	549 (88%)	1.24 (0.72,2.14)
Sport	Neither agree or disagree	18 (14%)	108 (86%)	1.00
	(Strongly) disagree	7 (10%)	60 (90%)	1.43 (0.57,3.62)
	(Strongly) agree	79 (13%)	553 (88%)	1.17 (0.67,2.03)
Being overweight/obese	Neither agree or disagree	0 (0%)	3 (100%)	- - -
	(Strongly) disagree	0 (0%)	3 (100%)	- - -
	(Strongly) agree	103 (13%)	719 (88%)	- - -
Manual work	Neither agree or disagree	18 (11%)	144 (89%)	1.00
	(Strongly) disagree	9 (15%)	53 (86%)	0.74 (0.31,1.74)
	(Strongly) agree	77 (13%)	527 (87%)	0.86 (0.50,1.48)

*CI* confidence interval, *OA* osteoarthritis

There was no significant difference in exercise use according to agreement with the statement ‘*exercise works just as well for everybody regardless of the amount of pain they have*’ (Table 5).

#### Beliefs about consequences: knowledge about risks/safety of exercises

Exercise use was significantly greater among GPs who agreed that quadriceps strengthening (OR 2.01 (1.29,3.15)) and general exercises (OR 1.99 (1.21,3.28)) are safe for everybody to do (Table 5). Exploration of the impact of treatment orientation, demonstrated a statistically significant greater exercise use among those in the top quartile on the behavioural subscale (173/193 (90%); OR 1.87 (1.03,3.39)), compared with those in the bottom quartile (153/186 (82%)). No statistically significant difference in exercise use was identified according to biomedical treatment orientation, however, exercise use was lower (162/194 (84%); OR 0.59 (0.33,1.08)) among GPs with the top quartile of biomedical scores compared to those in the bottom quartile (171/191 (90%)). No significant difference in exercise use was observed between those who had experienced uncertainty about the safety of exercise as a barrier (14/17 (82%); OR 0.67 (0.19,2.38)) when compared to those who had not (715/818 (87%)).

#### Moral norm

Exercise use was significantly associated with agreeing that local (OR 3.08 (1.96,4.83)) and general exercises (OR 2.63 (1.45,4.76)) should be prescribed to every patient with CKP (Table 5). However, disagreement with these statements was not significantly associated with exercise use.

#### Beliefs about capabilities: GP-related factors

Exercise use was significantly lower among GPs who reported previously experiencing uncertainty about the most appropriate exercise to recommend (170/210 (78%) versus 559/618 (91%); OR 0.38 (0.25,0.58)) and insufficient expertise to give detailed information (279/337 (83%) versus 450/497 (91%); OR 0.50 (0.33,0.76)), when compared to GPs who did not report experience of these barriers. Although exercise use was higher among GPs disagreeing that exercise for CKP is more effectively provided by physiotherapists than GPs (51/54 (94%); OR 2.05 (0.57,7.45)) and lower among GPs agreeing with this statement (553/640 (86%); OR 0.77 (0.42,1.40)), compared to those who neither disagreed nor agreed (116/130 (89%)), this association was not statistically significant.

#### Beliefs about capabilities: service-related factors

Beliefs about having enough time to manage patients with CKP were not significantly associated with exercise use; 427/491 (87%) of those agreeing with this statement versus 168/195 (86%) of those neither disagreeing nor agreeing (OR 1.07 (0.66,1.74)), and 132/145 (91%) of those disagreeing (OR 1.63 (0.81,3.29)). Similarly, there was no significant difference in exercise use among those agreeing (584/674 (87%); OR 0.67 (0.30,1.50)) or disagreeing (68/75 (91%); OR 1.00 (0.33,3.01)) that time constraints prevent GPs from providing advice on individual exercises for CKP when compared to those who neither disagreed nor agreed (68/75 (91%)). Exercise use among those agreeing (87%; OR 0.69 (0.34,1.42)) and disagreeing (92%; OR 1.17 (0.34,4.00)) that exercise for CKP would be used more frequently if access to physiotherapy was easier, was also not significantly different to that among GPs who neither disagreed nor agreed (90%). Finally, no significant difference was identified in exercise use among those reporting experience of insufficient time in consultations as a barrier (368/419 (88%); OR 1.10 (0.73,1.65)) when compared to those who had not experienced this (361/416 (87%)), nor among those reporting experience of difficulty accessing physiotherapy (235/273 (86%); OR 0.85 (0.56,1.30)) compared to those who had not experienced this (494/562 (88%)).

Although perceived time constraints were not associated with GPs’ exercise use, time constraints were highlighted as a prominent issue for GPs. Only 59% of all responding GPs agreed that they have enough time to manage patients with CKP, 82% agreed that time constraints prevent GPs from providing advice on individual exercises for CKP and 51% of the 815 GPs who reported having previously experienced barriers to using exercise highlighted insufficient time available in consultations as a barrier. It was therefore hypothesised that perceived time limitations may not impact *if* GPs used exercise but *how* they use it. A *posteriori* analysis identified that while use of exercise leaflets or exercise referrals were not associated with beliefs about time constraints, GPs who disagreed there were time limitations were more likely to suggest general (OR 2.17 (1.04,4.55)) or demonstrate local (OR 2.16 (1.06,4.42)) exercises to the patient described in the case vignette (see Additional file 4).

#### Beliefs about capabilities: patient-related factors

Exercise use among GPs who reported the experience of patients preferring other management options to exercise (261/291 (90%); OR 1.41 (0.90,2.21)) was similar to that among GPS who did not report experience of this barrier (468/544 (86%)). There was also no significant difference in exercise use among those who had experienced exercise not matching patients’ needs and/or expectations as a barrier (20/23 (87%); OR 0.97 (0.28,3.31)), compared with those who had not (709/801 (87%)).

#### Characteristics of the GPs

Exercise use was slightly lower among male (341/401 (85%; OR 0.64 (0.42,0.97)), compared with female (375/417 (90%)), GPs. Further, all fifty GPs who stated that they had a special interest in musculoskeletal conditions reported using exercise, compared with 87% of those who had no special interest in musculoskeletal conditions (Pearson Chi-square = 7.694, df1, *p* = 0.006). No other GP characteristics (type of GP, number of GPs in the practice, practice type, postgraduate training in musculoskeletal conditions and personal experience of CKP) were associated with exercise use.

#### Social influences

Of the barriers that GPs had previously experienced in using exercise for CKP, only 10/807 (1%) GPs reported these including GP colleagues not using or valuing exercise. No significant difference in exercise use was observed between those who had experienced this barrier (10/10 (100%)) and those who had not (719/825 (87%), Fisher’s Exact Test *p* = 0.624)).

#### Intention

No significant difference was found in exercise use among GPs who agreed (302/335 (90%); OR 1.58 (1.00,2.50)) and those who disagreed (111/128 (87%); OR 1.13 (0.63,2.03)) that managing patients with CKP is of clinical interest to them compared with those who neither disagreed nor agreed (313/367 (85%)). Although exercise use was higher among those who agreed that managing patients with CKP was a priority for them (200/221 (91%); OR 1.39 (0.82,2.35)) and lower among those who disagreed (109/131 (83%); OR 0.72 (0.43,1.23)), this was not statistically significant when compared with those who neither disagreed nor agreed (418/479 (87%)).

#### Habit or past behaviour

Habit or past behaviour was not systematically assessed. However, some results did indicate that previous clinical behaviour might influence the current behaviour of GPs, particularly with regards to use of the term ‘wear and tear’ (one GP responded ‘*I know this is no longer advised [as an] explanation but I can’t stop myself…*’) and the use of knee x-rays to investigate the patient (one respondent said ‘*Hard to drop this habit – research suggests is poor*’).

## Discussion

Using an analysis framework developed from sociocognitive theories, a cross-sectional questionnaire survey investigated a wide range of factors that potentially influence GPs’ exercise use for CKP. This study is the first known to the authors to concurrently investigate GPs’ attitudes, beliefs and behaviours specifically about exercise use for CKP. The factors most strongly associated with GPs’ exercise use were their beliefs about their role and about consequences, moral norm and GP-related beliefs about capabilities. Few GP characteristics were associated with, and no patient- or service-related beliefs about capabilities seemed to influence, GPs’ exercise use.

GPs’ perceptions of their role in initiating exercise into the management of a patient with CKP was clearly associated with their exercise use. The greater the GPs’ perceived role, the more likely they were to report using exercise. This finding is not novel, previously, Lipworth et at identified that interventions were more likely to be adopted if they were perceived to be aligned with the HCPs’ role [49]. Linked to perceptions about role, is moral norm (i.e. GPs’ perceptions of their responsibilities) [12, 28]. Respondents who agreed that GPs should prescribe local and general exercise to every patient with CKP were more likely to report using exercise. This may indicate that clarifying roles and responsibilities of GPs regarding the initiation of exercise for CKP may help to promote its use. The value of combining the three sociocognitive theories was demonstrated by the associations noted within the factor ‘*beliefs about consequences*’. While this is contained within both Godin [12] and Michie’s models [35], the addition of ‘*awareness of guidelines*’ from Pathman’s model [36] led to identification of a further, significantly associated factor; that exercise use was higher among those who had read recent relevant guidelines (NICE OA guidelines) [1]. This is despite previous findings that fewer than half of GPs who had read the guidelines felt it had changed their practice [50]. The associations identified between exercise use and perceived roles, moral norm and awareness of guidelines may present a potential problem with optimising GPs’ management of CKP, as GPs’ roles with regards to initiating exercise and following-up patients to ensure continuation with exercise are not currently clearly defined, only a third of responding GPs, which are likely to represent the most interested among the sample, had read the NICE OA guidelines and only 58% GPs reported having read the same NICE OA guidelines in a previous questionnaire survey [50].

Further value of the analysis framework was demonstrated through the TDF-prompted inclusion of explicit GP-, service- and patient-related factors within *‘beliefs about capabilities’*. This resulted in the identification that only GP-related factors, such as uncertainty of the most appropriate type of exercise to use and insufficient expertise to give detailed exercise information, had a significant influence on the GPs’ exercise use. External factors such as service design (e.g. time constraints, access to physiotherapy) and patients’ treatment preferences did not impact GPs’ exercise use in the same way. This was interesting given the pervasive perception of time limitations and that one-third of GPs believed that patients preferred other management options. Further, there was little or no association identified among disease-related factors such as beliefs about symptom or damage severity and perceived risk factors. This, combined with the strong influence of beliefs about role, and significant associations between moral norm and beliefs about efficacy, may suggest that internal factors arising from GPs are more influential than external factors acting on GPs.

Some factors within the analysis framework were not associated with behaviour in ways that would have been expected. For example, when agreement with attitude statements was significantly associated with behaviour, a reciprocal effect was not seen among those who disagreed (and vice versa). Further, exercise use was similar among GPs who held negative beliefs about its safety or efficacy and those who held positive beliefs, with the lowest exercise use observed among GPs who neither disagreed nor agreed (Table 5). The most likely explanation for this finding is that uncertainty is a barrier to GPs’ exercise use.

### Strengths and limitations

The primary strength of this study is the concurrent, theory-based, investigation of attitudes, beliefs and behaviours of GPs regarding exercise for CKP, which has enabled exploration of the key influences on GPs’ clinical behaviours in this context. The national sample mitigates against the confounding effects of local service anomalies. The value of combining multiple theories to more comprehensively assess relevant behavioural influences has previously been highlighted [51]. Thus, using the analysis framework comprised of a combination of three sociocoginitive theories resulted in greater breadth in the factors considered as well as acknowledgement of a directional relationship between factors. While it is accepted that the exact placement of certain factors within the analysis framework could be debatable, as some may fit in multiple positions, single positions are given to each factor to maximise clarity while maintaining the key content.

The low response is a clear limitation and, consequently, GPs’ exercise use may be over-estimated. This may have also impacted analyses of associations with exercise use by homogenising the sample. Thus significant associations may have been masked by small numbers in some subgroups. Despite the number of GPs returning questionnaires exceeding the 748 calculated to be required, the survey was underpowered to confidently detect a difference in exercise use (which was smaller than anticipated) according to treatment orientation. It is acknowledged that multiple testing can result in identification of statistically significant results by chance, thus, in this exploration of potentially associated factors, patterns of multiple significant associations within each factor of the analysis framework were considered. It is also noted that while some of the observed associations are statistically significant, the absolute difference in exercise use between compared groups is small in some cases. In retrospect, certain factors within the analysis framework could have been strengthened and given greater focus within the survey. For example, there was insufficient direct data to adequately assess the association between patient-related social norms and habit/past behaviours on GP behaviours and uncertainty was insufficiently conceptualised within beliefs about consequences. Lack of explicit focus on uncertainty may be a significant omission as previous work has identified that GPs experience more uncertainty relating to guidelines than other medical specialty doctors [15]. Finally, the proportion of GPs who were partners (79%) in this study is slightly in excess of that among the GP population (~75%) in England [52]. It is possible that by nature, partners remain in the same practice for longer and thus Binley’s database for contact details may be more accurate for GP partners. It is unlikely that this small difference will have had a significant effect on the generalisability of the results.

### Implications

**Fig. 2:**
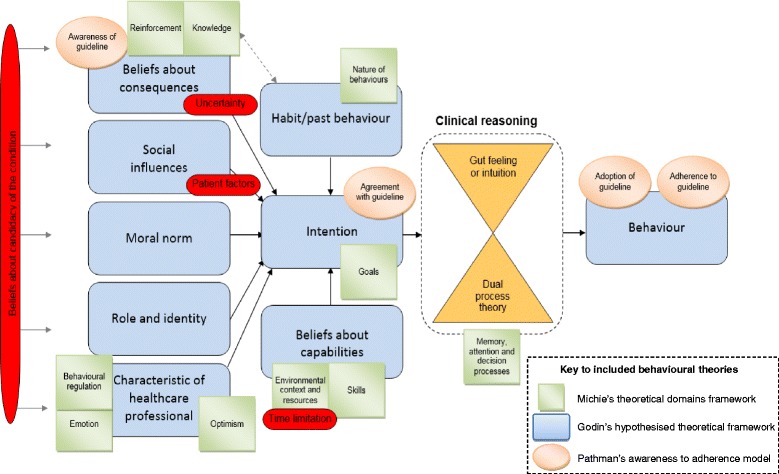
Revised analysis framework

The results obtained from this work can be used to inform future interventions targeted at optimising GPs’ exercise use for patients with CKP. For example, these findings imply that addressing the factors that are closest to the GP, such as promoting their belief that it is their role and responsibility, that exercise can be used for all patients and that it is effective and safe, and through equipping GPs with pragmatic approaches to implement this, may enhance their use of exercise for CKP. However, further refinement of approaches used to investigate GP behaviours would be beneficial in order to better understand the identified anomalies between the beliefs and the behaviours of GPs. A shift in the focus from sociocognitive behavioural theories to factors which impact clinical reasoning [53], specifically regarding decision-making theory, may be required. One such theory is the dual process theory (DPT), which describes two types of decision-making: system 1, a fast, intuitive, automatic approach which is not cognitively demanding but can be inaccurate, and system 2, a slow, conscious, analytical approach [53–55]. When faced with time pressures, lack of confidence or problems that are perceived to be routine or certain, clinicians may favour system 1 decision-making [53]. This might explain why GPs use exercise despite uncertainties about its safety or efficacy. For example, GPs may recommend exercise because they know they should (without considering the reasons), but only when asked to consider this in a more analytical way (i.e. within a survey or when questioned by a patient), do their uncertainties arise. A revised analysis framework is presented in Fig. 2. This continues to draw on the same three theoretical models explaining the associations between attitudes and behaviours: Godin’s hypothesised theoretical framework [12], Pathman’s awareness-to-adherence model [36] and the (now updated) TDF [56]. For the aforementioned reasons, the revised framework includes focus on clinical reasoning and places greater emphasis on the impact of patient’s requests, preferences and traits (potentially relevant patient factors are provided in the hexagons in the expanded patient factors box within Fig. 2), perceptions of time limitations, the potential impact of uncertainty on GPs’ behaviours and it highlights the potential impact of habit/past behaviour. Further, given the low response is likely to have biased results to more accurately reflect the attitudes, beliefs and behaviours of the most interested GPs, it is uncertain whether GPs may even enter the processes outlined by the framework if they normalise CKP to the extent of believing that it is not eligible for medical treatment (i.e. negative beliefs about candidacy); thus the role of candidacy has been added to the beginning of the framework for future consideration. Undertaking research to validate and further refine this framework, for example, by identifying a differential weighting for the different factors contained within it, would be of value.

## Conclusion

This survey has identified the factors within a sociocognitive-derived analysis framework that are associated with GPs’ exercise use for patients with CKP, these are factors which are closest to the GP, such as: perceived role (i.e. the greater the perceived GP role in initiating exercise, the greater the likelihood of using exercise), beliefs about consequences (i.e. agreement that exercise will improve CKP), moral norm (i.e. believing that GPs should prescribe exercise to all patients) and GP-related belief about capabilities (i.e. perception of own expertise). The more external service- (i.e. time limitations and access to physiotherapy), patient- (i.e. patient preferences) and disease-related (i.e. symptom and damage severity, risk factors) factors appeared to be less influential on GPs’ exercise use. These results suggest that interventions which promote GPs’ perceptions of having a clear role in, and responsibility for, initiating exercise with every patient with CKP and the belief that exercise is beneficial may help to optimise GPs’ behaviours. However not all results were sufficiently explained by the analysis framework. A greater focus on clinical reasoning, in particular factors which influence decision-making, and inclusion of the impact of uncertainty and patient factors in future approaches used to investigate behaviours may be necessary to best understand the influences on GPs’ behaviours and should be tested in future work.

## Additional files

Additional file 1: Management of Chronic Knee Pain Study Questionnaire. (PDF 381 kb)
Additional file 2: Outline of how questionnaire content maps onto underpinning model. (DOCX 32 kb)
Additional file 3: Summary of associations between elements of the analysis framework and GPs’ use of exercise. (DOCX 20 kb)
Additional file 4: Association between perception of time constraints and delivery method for exercises. (DOCX 16 kb)

## Abbreviations

CKP: Chronic knee pain
DPT: Dual process theory
GP: General practitioner
HCP: Healthcare professional
LBP: Low back pain
MDS: Minimum data sets
OA: Osteoarthritis
PABS_PT: Pain Attitudes and Beliefs Scale for Physiotherapists
TDF: Theoretical domains framework
TPB: Theory of planned behaviour
