# Supplementary material for: What influences general practitioners’ use of exercise for patients with chronic knee pain? Results from a national survey
Source: BMC Fam Pract. 2016 Dec 19;17:172. doi: 10.1186/s12875-016-0570-4 (PMC5168590; doi:10.1186/s12875-016-0570-4)
Supplement: Additional file 1: — Management of Chronic Knee Pain Study Questionnaire. (PDF 381 kb) [file 12875_2016_570_MOESM1_ESM.pdf]

Arthritis Research UK | primary care centre

Keele University

Management of Chronic Knee Pain Study

We are seeking the views of general practitioners who treat patients over 45 years old, with chronic knee pain.

If you **are not** a general practitioner, please tick this box and return the questionnaire without completing it any further ☐

If you **have not** managed someone with chronic knee pain in the last 6 months, please tick this box and return the questionnaire without completing it any further. ☐

This questionnaire should take no more than **20 minutes** to complete.

Return of your completed questionnaire will be interpreted as you providing your consent to participate in this study.

If you would like to participate in this study please either:

- Complete this paper version of the questionnaire and return it to Dr Elizabeth Cottrell, Academic Clinical Fellow GP Specialty Trainee, at the Arthritis Research UK Primary Care Centre, Keele University, Staffordshire, ST5 5BG using the enclosed FREEPOST envelope.
  OR
- Complete the online version of this questionnaire accessible at https://www.surveymonkey.com/s/Management_CKP

If you have any questions about this questionnaire or the study in general you can email Dr Elizabeth Cottrell at e.cottrell@keele.ac.uk

Instructions for completing this questionnaire

- When completing the questionnaire, please try and provide answers that most accurately reflect your usual clinical practice. There are no ‘correct’ or ‘incorrect’ answers.
- Where relevant please answer questions by ticking a box e.g. ☑
- Please do not consult any literature while completing this questionnaire.

Thank you for your help with this study

Unique survey ID	

Section 1: About you

1.1 Please state the year in which you qualified as a General Practitioner ☐

1.2 How many General Practitioners work in your practice (including yourself)? ☐

1.3 How do you best **describe yourself** (please tick **one box** only)

☐ GP Partner ☐ Salaried GP ☐ Locum GP

☐ Other, please specify ____________________

1.4 Is your practice ☐ Urban ☐ Semi-rural ☐ Rural

1.5 Are you ☐ Male ☐ Female

1.6 Are you a **GP with a special interest (GPwSI)** in musculoskeletal conditions?

☐ Yes ☐ No

1.7 Do you remember receiving any specific postgraduate musculoskeletal training which contained education about chronic knee pain? (By this we <u>do not</u> mean clinical placements or jobs in rheumatology or orthopaedics)

☐ Yes ☐ No

1.8 Do you have, or have you ever suffered from chronic knee pain **yourself**?

☐ Yes ☐ No

Section 2: Clinical scenario of a patient with chronic knee pain

Presented below is a clinical scenario of a patient **with chronic knee pain** who presents to you with this problem for the first time. All questions that follow relate to the care you would give this particular patient. Think about the patient's first consultation with you.

Patient: Mrs Jones, 58-year-old Prison Officer

History: First presentation of gradually worsening bilateral knee pain (right worse than left) over 2 years
No history of trauma
Pain always present when walking and at rest, worst when climbing stairs. No night pain.
Managing activities of daily living. Difficulty gardening.
Stopped going to gym – thinks was making pain worse
Only treatment tried is Ibuprofen once or twice when pain "really bad" – no benefit.
Came today finding work increasingly difficult due to the stairs
Usually well – no comorbidities

Medication: Nil

Examination: Body Mass Index 33
Knees – bilaterally no effusions. Joint tenderness upon palpation. Bilateral coarse crepitations.
Slightly reduced flexion of the right knee.
Hips – no abnormality detected

2.1 What **diagnosis** would you make at this point?

____

2.2 Using the words you would use with the patient, ***briefly*** state how you would **describe your diagnosis** to the patient

____

____

____

2.3 The patient's **symptoms** are: (please tick the **one** box that best reflects your opinion)

☐ Very severe ☐ Severe ☐ Moderate ☐ Mild ☐ Very mild

2.4 It is most likely that this patient's symptoms result from **knee damage** that is: (please tick the **one** box that best reflects your opinion)

☐ Very severe ☐ Severe ☐ Moderate ☐ Mild ☐ Very mild

2.5 Using the words you would use with the patient, ***briefly*** describe **what the future is likely to hold** with regards to her knee problem

____

____

2.6 What **investigation(s)/assessment(s)** would you do/order for this patient **at this point** and for what reason?

	Please indicate the reason for choosing this investigation (tick all that apply)					
Please tick **all** you would undertake	Reassure patient	To meet referral criteria	Confirm diagnosis	Inform treatment	Rule out other diagnoses	Inform prognosis
☐ None	---	---	---	---	---	---
☐ Blood tests	☐	☐	☐	☐	☐	☐
☐ Oxford knee score	☐	☐	☐	☐	☐	☐
☐ Knee x-ray	☐	☐	☐	☐	☐	☐
☐ Other - please state __________	☐	☐	☐	☐	☐	☐

2.7 **At this consultation**, what approaches would you use, or suggest, to manage this patient? (please tick **all** that apply)

☐ Ice
☐ Heat
☐ Acupuncture
☐ Rest
☐ Weight loss
☐ None
☐ Keep active
☐ Provision of walking stick(s)
☐ Advice on footwear
☐ Exercise
☐ Bed rest
☐ Transcutaneous electrical nerve stimulation
☐ Weak opioids (e.g. codeine)
☐ COX II inhibitor
☐ Antidepressants
☐ Injection of steroids
☐ Oral non-selective NSAIDs (e.g. Ibuprofen)
☐ Topical NSAID
☐ Paracetamol
☐ Glucosamine/ chondroitin
☐ Topical capsaicin
☐ Strong opioids (e.g. morphine, Tramadol)

☐ Other (please state)

____

If you ticked "Exercise" in question 2.7 please go on to answer question 2.8. If not, please move straight on to question 2.9

2.8 There are a variety of approaches that can be used to manage chronic knee pain in general practice.

Of the following different approaches, **which, if any, would you use for this patient at this point**?

a) General exercises or increasing physical activity

I would use ☐ ⇨ If you ***would use*** this strategy, please give details about what you would **actually do** (please tick any that apply)

- ☐ Suggest general exercises ⇨ Please state type of exercise ____________________
- ☐ Give a leaflet
- ☐ Refer
- ☐ Other ⇨ Please state what other actions you would do ____________________

I would **not** use ☐ ⇨ If you ***would not use*** the strategy ***but would like to***, please tell us **what prevents** you (please tick any that apply)

- ☐ Insufficient expertise
- ☐ Insufficient time
- ☐ Other ⇨ Please state what other reasons prevent you (e.g. patient factors, access difficulties, uncertainty of benefit etc) ____________________

b) Local knee or quadriceps strengthening exercises

I would use ☐ ⇨ If you ***would use*** this strategy***,*** please give details about what you would **actually do** (please tick any that apply)

- ☐ Demonstrate specific exercises
- ☐ Give a leaflet
- ☐ Refer
- ☐ Other ⇨ Please state what other actions you would do ____________________

I would **not** use ☐ ⇨ If you ***would not use*** the strategy ***but would like to***, please tell us **what prevents** you (please tick any that apply)

- ☐ Insufficient expertise
- ☐ Insufficient time
- ☐ Other ⇨ Please state what other reasons prevent you (e.g. patient factors, access difficulties, uncertainty of benefit etc) ____________________

c) Follow up patient to check to see if she is undertaking exercise on a regular basis

I would use ☐ ⇨ If you ***would use*** this strategy***,*** please give details about what you would **actually do** (please tick any that apply)

- ☐ Planned follow-up ⇨
  - When: Please define number of days/weeks/months ____________
  - How: ☐ Face to face ☐ Via telephone
  - With whom: ☐ Yourself ☐ Another professional or service – please state ____________
- ☐ Opportunistic follow-up ⇨ When: ☐ If patient fails to improve ☐ Check when patient is next seen with this or any problem
- ☐ Other ⇨ Please state what other actions you would do ____________________

I would **not** use ☐ ⇨ If you ***would not use*** the strategy ***but would like to***, please tell us **what prevents** you (please tick any that apply)

- ☐ Insufficient expertise
- ☐ Insufficient time
- ☐ Other ⇨ Please state what other reasons prevent you ____________________

2.9	Would you refer the patient to see someone else, either in the primary or community team or into secondary care, at this point?<br><br>If yes, to whom would you refer her?<br><br>________________________________	☐	Yes	☐	No
2.10	Do you usually provide **written information** for patients in this situation?	☐	Yes	☐	No

If yes, please state the source of your written information

☐ Patient.co.uk or Emis Mentor ☐ Arthritis Research UK (previously known as ARC) or Arthritis Care ☐ Other, please state ________________

If you would provide written information, it would be very helpful to see a copy by returning it in the freepost envelope provided (**please write on your unique survey ID**) or by providing us with the link for any online information you access.

We are interested in your clinical opinion about patients **aged 45 years and over** with **chronic knee pain**. In this age group chronic knee pain is almost always due to knee osteoarthritis. Please answer all of the following questions using the definition of chronic knee pain as follows: knee pain and associated symptoms that have been present for more than 3 months not resulting from a fracture, infection, systemic rheumatological problem, metastases or surgery.

Section 3: Chronic knee pain in general practice

		I have never heard about or read it	I have heard of it but not seen it	I have seen it but not read it	I have read the full guideline and/or summary	I have read and consider the guideline when planning management
3.1	How much have you heard about or read the guideline published by NICE in 2008 for the care and management of osteoarthritis in adults? (please tick **one** box)	☐	☐	☐	☐	☐

Please consider **your role** in managing patients aged over 45 years old with chronic knee pain. Please indicate the extent to which you agree or disagree with the statements given by ticking **one** box per row.

		Strongly disagree	Disagree	Neither agree or disagree	Agree	Strongly agree
3.2	It is part of my job to manage people with chronic knee pain	☐	☐	☐	☐	☐
3.3	I have enough time to manage patients with chronic knee pain	☐	☐	☐	☐	☐
3.4	Managing patients with chronic knee pain is a priority for me	☐	☐	☐	☐	☐
3.5	Managing patients with chronic knee pain is of clinical interest to me	☐	☐	☐	☐	☐
3.6	It is part of my job to reassure patients about the safety of exercise for chronic knee pain	☐	☐	☐	☐	☐
3.7	It is part of my job to provide patients with chronic knee pain with a written management plan	☐	☐	☐	☐	☐

		I have no role in including exercise in the management	I inform patients that exercise is a management option	I advise patients to use exercise to manage their knee pain	I recommend the types of exercise patients could use	I give information on the type, frequency and duration of specific exercises
3.8	Which statement best describes your role in including exercise in the management plan of a patient with chronic knee pain? (please tick **one** box)	☐	☐	☐	☐	☐

3.9 We are interested to hear about **your experiences of barriers which might prevent the use of exercise** in your management of chronic knee pain. Please tick **all** of the barriers that you have experienced

- ☐ Insufficient time in consultations
- ☐ Insufficient expertise to give detailed information
- ☐ Difficulty accessing physiotherapy
- ☐ Uncertainty about the effects of exercise
- ☐ Patients prefer other management options
- ☐ My GP colleagues do not use or value exercise
- ☐ Uncertainty about the most appropriate type of exercise
- ☐ Uncertainty about the safety of exercise
- ☐ Other (please state) ____

____

Section 4: Your views about chronic knee pain

Below is a list of **possible causes** for a patient developing chronic knee pain. Please indicate the extent to which you agree or disagree with these causes by ticking **one** box in each row.

		Strongly disagree	Disagree	Neither agree or disagree	Agree	Strongly agree
4.1	Hereditary/runs in the family	☐	☐	☐	☐	☐
4.2	Being overweight/obese	☐	☐	☐	☐	☐
4.3	A person's own mental attitude e.g. thinking about life negatively	☐	☐	☐	☐	☐
4.4	A person's emotional state e.g. feeling down, anxious	☐	☐	☐	☐	☐
4.5	Ageing	☐	☐	☐	☐	☐
4.6	Accident or injury	☐	☐	☐	☐	☐
4.7	Manual work	☐	☐	☐	☐	☐
4.8	Sport	☐	☐	☐	☐	☐
4.9	Osteoarthritis	☐	☐	☐	☐	☐
4.10	Changes consistent with osteoarthritis seen on x-ray	☐	☐	☐	☐	☐

When completing the following questions, please consider patients **aged over 45 years old** with **chronic knee pain**. Please indicate the extent to which you agree or disagree with the statements given by ticking one box per row.

		**Totally disagree**	**Largely disagree**	**Disagree to some extent**	**Agree to some extent**	**Largely agree**	**Totally agree**
4.11	Mental stress can cause chronic knee pain even in the absence of tissue damage	☐	☐	☐	☐	☐	☐
4.12	The cause of chronic knee pain is unknown	☐	☐	☐	☐	☐	☐
4.13	Pain is a nociceptive stimulus, indicating tissue damage	☐	☐	☐	☐	☐	☐
4.14	A patient suffering from severe chronic knee pain will benefit from physical exercise	☐	☐	☐	☐	☐	☐
4.15	Functional limitations associated with chronic knee pain are the result of psychosocial factors	☐	☐	☐	☐	☐	☐
4.16	Patients with chronic knee pain should preferably practice only pain free movements	☐	☐	☐	☐	☐	☐
4.17	Therapy may have been successful even if pain remains	☐	☐	☐	☐	☐	☐
4.18	Chronic knee pain indicates the presence of organic injury	☐	☐	☐	☐	☐	☐
4.19	If chronic knee pain increases in severity, I immediately adjust the intensity of my treatment accordingly	☐	☐	☐	☐	☐	☐
4.20	If therapy does not result in a reduction in chronic knee pain, there is a high risk of severe restrictions in the long term	☐	☐	☐	☐	☐	☐
4.21	Pain reduction is a precondition for the restoration of normal functioning	☐	☐	☐	☐	☐	☐
4.22	Increased pain indicates new tissue damage or the spread of existing damage	☐	☐	☐	☐	☐	☐
4.23	There is no effective treatment to eliminate chronic knee pain	☐	☐	☐	☐	☐	☐
4.24	Even if the pain has worsened, the intensity of the next treatment can be increased	☐	☐	☐	☐	☐	☐
4.25	If patients complain of pain during exercise, I worry that damage is being caused	☐	☐	☐	☐	☐	☐
4.26	The severity of tissue damage determines the level of pain	☐	☐	☐	☐	☐	☐
4.27	Learning to cope with stress promotes recovery from chronic knee pain	☐	☐	☐	☐	☐	☐
4.28	Exercises that may be knee straining should <u>not</u> be avoided	☐	☐	☐	☐	☐	☐
4.29	In the long run, patients with chronic knee pain have a higher risk of developing severe functional impairments	☐	☐	☐	☐	☐	☐

Section 5: Your views about the role of exercise in treating chronic knee pain

We are interested in your views about the **role of exercise** in the treatment of **chronic knee pain in patients over 45 years old**. Please indicate the extent to which you agree or disagree with the statements given by ticking one box per row.

		Strongly disagree	Disagree	Neither agree or disagree	Agree	Strongly agree
5.1	GPs should prescribe quadriceps strengthening exercises to every patient with chronic knee pain	☐	☐	☐	☐	☐
5.2	GPs should prescribe general exercise, for example, walking or swimming, for every patient with chronic knee pain	☐	☐	☐	☐	☐
5.3	Knee problems are improved by quadriceps strengthening exercises	☐	☐	☐	☐	☐
5.4	Knee problems are improved by general exercise, for example walking or swimming	☐	☐	☐	☐	☐
5.5	Quadriceps strengthening exercises for the knee are safe for everybody to do	☐	☐	☐	☐	☐
5.6	General exercise, for example walking or swimming is safe for everybody to do	☐	☐	☐	☐	☐
5.7	Exercise for chronic knee pain is most beneficial when it is tailored to meet individual patient needs	☐	☐	☐	☐	☐
5.8	A standard set of exercises is sufficient for every patient with chronic knee problems	☐	☐	☐	☐	☐
5.9	GPs should educate chronic knee pain patients about how to change their lifestyle for the better	☐	☐	☐	☐	☐
5.10	It is important that people with chronic knee pain increase their overall activity levels	☐	☐	☐	☐	☐
5.11	How well a patient complies with their exercise programme determines how effective it will be	☐	☐	☐	☐	☐
5.12	GPs should follow up patients to monitor extent of continuation of exercises	☐	☐	☐	☐	☐
5.13	It is the patient's own responsibility to continue doing their exercise programme	☐	☐	☐	☐	☐
5.14	Exercise is effective for patients if an x-ray shows severe knee osteoarthritis	☐	☐	☐	☐	☐
5.15	Exercise works just as well for everybody, regardless of the amount of pain they have	☐	☐	☐	☐	☐
5.16	Increasing the strength of the muscles around the knee stops the knee problem getting worse	☐	☐	☐	☐	☐
5.17	Increasing overall activity levels stops the knee problem getting worse	☐	☐	☐	☐	☐
5.18	Exercise for chronic knee pain is more effectively provided by physiotherapists than GPs	☐	☐	☐	☐	☐
5.19	Time constraints prevent GPs from providing advice on individual exercises for chronic knee pain	☐	☐	☐	☐	☐
5.20	Exercise for chronic knee pain should preferably be used after drug treatment has been tried	☐	☐	☐	☐	☐
5.21	Exercise for chronic knee pain would be used more frequently if access to physiotherapy was easier	☐	☐	☐	☐	☐

Would you be happy for us to contact you again in the future regarding this study?

☐ Yes ☐ No

If you answered YES to the question above please provide your name and contact details below (these details will be kept separately from your responses to the questionnaire):

**Name:**	
**Daytime telephone number:**	
**Address:**	
**Email:**	

End of Questionnaire

You have reached the end of the questionnaire. Please return the questionnaire in the **FREEPOST** envelope provided.

If you have any questions about this questionnaire or the study in general, you can email Dr Elizabeth Cottrell at e.cottrell@keele.ac.uk

Thank you for taking the time to complete this questionnaire. Your time and participation is greatly appreciated.

Unique survey ID
