# Supplementary material for: What influences general practitioners’ use of exercise for patients with chronic knee pain? Results from a national survey
Source: BMC Fam Pract. 2016 Dec 19;17:172. doi: 10.1186/s12875-016-0570-4 (PMC5168590; doi:10.1186/s12875-016-0570-4)
Supplement: Additional file 2: — Outline of how questionnaire content maps onto underpinning model. (DOCX 32 kb) [file 12875_2016_570_MOESM2_ESM.docx]

**Additional File 2 Outline of how questionnaire content maps onto underpinning model**

| Element analysis framework |  | Item(s) in study tool relating to this factor |
| --- | --- | --- |
| Beliefs about consequences |  |  |
|  | Knowledge and attitudes about the efficacy of exercise | Q5.3 Knee problems are improved by quadriceps strengthening exercises  Q5.4 Knee problems are improved by general exercise, for example walking or swimming  Q5.16 Increasing the strength of the muscles around the knee stops the knee problems getting worse  Q5.17 Increasing overall activity levels stops the knee problem getting worse  Q3.9 We are interested to hear about your experiences of barriers which may prevent the use of exercise in the management of CKP: uncertainty about the effects of exercise |
|  | Awareness of management recommendations | Q3.1 How much have you heard about or read the guideline published by NICE in 2008 for the care and management of osteoarthritis in adults?  Q5.7 Exercise for CKP is most beneficial when it is tailored to meet individual patient needs  Q5.8 A standard set of exercises is sufficient for every patient with CKP  Q5.10 It is important that people with CKP increase their overall activity levels  Q5.11 How well a patient complies with their exercise programme determines how effective it will be  Q5.20 Exercise for CKP should preferably be used after drug treatment has been tried |
|  | Factors that may be perceived to influence efficacy of exercise | Q2.1 What diagnosis would you make at this point?  Q2.2 Using the words you would use with the patient, briefly state how you would describe your diagnosis to the patient  Q2.3 The patient’s symptoms are (very) severe, moderate, (very) mild  Q2.4 It is most likely that this patient’s symptoms result from knee damage that is (very) severe, moderate, (very) mild  Q2.5 Using the words you would use with the patient briefly describe what the future is likely to hold with regards to her knee problem  Q2.6 What investigations will you do/order for the patient at this point?  Q4.1-4.10 Possible causes of CKP  Q5.14 Exercise is effective for patients if an x-ray shows severe knee osteoarthritis  Q5.15 Exercise works just as well for everybody, regardless of the amount of pain they have |
|  | Knowledge and attitudes about risks/safety of exercises | Q3.9 We are interested to hear about your experiences of barriers which may prevent the use of exercise in the management of CKP: uncertainty about the safety of exercise  Q4.11-4.29 adapted PABS_PT items  Q5.5 Quadriceps strengthening exercises for the knee are safe for everybody to do  Q5.6 General exercise, for example walking or swimming is safe for everybody to do |
| Social influences | Q3.9 We are interested to hear about your experiences of barriers which may prevent the use of exercise in the management of CKP: my GP colleagues do not use or value exercise | |
| Moral norm | Q5.1 GPs should prescribe quadriceps strengthening exercises to every patient with CKP  Q5.2 GPs should prescribe general exercise, for example, walking or swimming, for every patient with CKP | |
| Role and identity | Q3.2 It is part of my job to manage people with CKP  Q3.6 It is part of my job to reassure patients about the safety of exercise for CKP  Q3.7 It is part of my job to provide patients with CKP with a written management plan  Q3.8 Which statement best describes your role in including exercise in the management plan of a patient with CKP?  Q5.9 GPs should educate CKP patients about how to change their lifestyle for the better  Q5.12 GPs should follow-up patients to monitor extent of continuation of exercises  Q5.13 It is the patient’s own responsibility to continue doing their exercise programme | |
| Characteristics of healthcare professional | Q1.1 Please state the year in which you qualified as a General Practitioner  Q1.2 How many GPs work in your practice?  Q1.3 How do you best describe yourself? (GP partner, salaried GP, locum GP, Other)  Q1.4 Is your practice urban? Semi-rural? Rural?  Q1.5 Are you male? Female?  Q1.6 Are you a GP with a special interest in musculoskeletal conditions?  Q1.7 Do you remember receiving any specific postgraduate musculoskeletal training which contained education about CKP?  Q1.8 Do you have, or have you ever suffered from chronic knee pain yourself? | |
| Beliefs about capabilities | GP-related factors | Q2.8 I would not use [general exercise, local exercise, or follow-up] [due to] insufficient expertise  Q3.9 We are interested to hear about your experiences of barriers which may prevent the use of exercise in the management of CKP: insufficient expertise to give detailed information, uncertainty about the most appropriate type of exercise  Q5.18 Exercise for CKP is only effectively provided by physiotherapists |
|  | Service-related factors | Q2.8 I would not use [general exercise, local exercise, or follow-up] [due to] insufficient time  Q3.3 I have enough time to manage patients with CKP  Q3.9 We are interested to hear about your experiences of barriers which may prevent the use of exercise in the management of CKP: insufficient time in consultations, difficulty accessing physiotherapy  Q5.19 Time constraints prevent GPs from providing advice on individual exercises for CKP  Q5.21 Exercise for CKP would be used more frequently if access to physiotherapy was easier |
|  | Patient related factors | Q3.9 We are interested to hear about your experiences of barriers which may prevent the use of exercise in the management of CKP: patients prefer other management options, exercise does not match patient needs and/or expectations |
| Intention | Motivation and goals | Q3.4 Managing patients with CKP is a priority for me  Q3.5 Managing patients with CKP is of clinical interest to me |
| Habit/past behaviour |  | Not formally examined within the questionnaire |
| Behaviour |  | Q2.7 At this consultation, what approaches would you use, or suggest, to manage this patient?  Q2.8 There are a variety of approaches that can be used to manage CKP in general practice. Of the following different approaches, which, if any, would you use for this patient at this point? General exercises or increasing physical activity, local knee or quadriceps strengthening exercises, follow-up to check to see if she is undertaking exercise on a regular basis.  Q2.9 Would you refer this patient to someone else, either in the primary or community team or into secondary care, at this point?  Q2.10 Do you usually provide written information for patients in this situation? |
| CKP = chronic knee pain; GP = general practitioner; NICE = National Institute of Health and Care Excellence | | |
