# Supplementary material for: What influences general practitioners’ use of exercise for patients with chronic knee pain? Results from a national survey
Source: BMC Fam Pract. 2016 Dec 19;17:172. doi: 10.1186/s12875-016-0570-4 (PMC5168590; doi:10.1186/s12875-016-0570-4)
Supplement: Additional file 3: — Summary of associations between elements of the analysis framework and GPs’ use of exercise. (DOCX 20 kb) [file 12875_2016_570_MOESM3_ESM.docx]

Additional File 3 Summary of associations between elements of the analysis framework and GPs’ use of exercise

| **Item(s) in study questionnaire** | | | **Significantly associated with exercise use** | | **Comment** | |  |
| --- | --- | --- | --- | --- | --- | --- | --- |
|  |  |  | **Odds ratio (95% CI) if significant** | |  |  |  |
| **ROLE AND IDENTITY** | | | | | | |  |
| Agreement that it is part of their job to manage people with CKP | | 🗶 | | | | Disagreement is not significantly associated* | |
| Agreement that GPs should educate CKP patients about how to change their lifestyle for the better | | 2.43 (1.22, 4.82)* | | | | Disagreement is not significantly associated* | |
| Agreement that it is part of their job to provide patients with CKP with a written management plan | | 2.21 (1.29, 3.80)* | | | | Disagreement is not significantly associated* | |
| Beliefs about the role of a GP in including exercise in the management plan of a patient with CKP | | 30.71 (5.02, 188.01)**  Give information on type, duration and frequency of exercise | | | | The greater the believed role, the greater the OR for the use of exercise | |
| Agreement that it is part of their role to reassure patients about the safety of exercise for CKP | | 3.57 (1.91, 6.59)* | | | | Disagreement is not significantly associated* | |
| Agreement that it is the patient’s own responsibility to continue doing their exercise programme | | 🗶 | | | | Disagreement is not significantly associated* | |
| Agreement that GPs should follow-up patients to monitor extent of continuation of exercises | | 🗶 | | | | Disagreement not significantly associated* | |
| **BELIEFS ABOUT CONSEQUENCES** | | | | | | |  |
| **Knowledge and attitudes about the efficacy of exercise** | | | | | | |  |
| Agreement that knee problems are improved by quadriceps strengthening exercises | | | 3.23 (1.94, 5.39)* | | Insufficient data to assess association with disagreement | |  |
| Agreement that knee problems are improved by general exercise | | | 2.63 (1.38, 5.02)* | | Insufficient data to assess association with disagreement | |  |
| Agreement that increasing the strength of the muscles around the knee stops the knee problems getting worse | | | 🗶 | | Disagreement is not significantly associated* | |  |
| Agreement that increasing overall activity levels stops the knee problem getting worse | | | 🗶 | | Disagreement is not significantly associated* | |  |
| Prior experience of being uncertain about the effects of exercise as a barrier to using exercise | | | 0.13 (0.07, 0.24) | |  | |  |
| **Awareness of management recommendations** | | | | | | |  |
| GP has read the NICE OA guideline | | | 1.68 (1.07, 2.64) | |  | |  |
| Agreement that exercise for CKP should preferably be used after drug treatment has been tried | | | 🗶 | | Disagreement associated with increased use of exercise (OR 2.10 (1.22-3.63) * | |  |
| Concurrent use of first- and second-line and not-recommended treatment approaches | | | 2.31 (1.19, 4.46)  First-line | | Concurrent use of second-line or not recommended approaches not associated | |  |
| Agreement that exercise for CKP is most beneficial when it is tailored to meet individual patient needs | | | 🗶 | | Insufficient data to assess association with disagreement | |  |
| Agreement that a standard set of exercises is sufficient for every patient with CKP | | | 🗶 | | Disagreement is not significantly associated* | |  |
| Agreement that it is important that people with CKP increase their overall activity levels | | | 2.18 (1.22, 3.91)* | | Disagreement is not significantly associated* | |  |
| Agreement that how well a patient complies with their exercise programme determines how effective it will be | | | 🗶 | | Disagreement associated with reduced use of exercise (OR 0.33 (0.11-0.96))* | |  |
| **Factors that may be perceived to influence efficacy of exercise** | | | | | | |  |
| GPs’ beliefs about the severity of the patient’s symptoms | | | 🗶 | | Trend towards increasing use of exercise if believe symptoms to be (very) mild** | |  |
| GPs’ beliefs about the severity of the underlying knee damage | | | Belief damage is moderate  2.24 (1.17, 4.29)** | | Trend towards increasing use of exercise if believe symptoms to be (very) mild** | |  |
| Use of the term wear and tear in the description of the diagnosis to the patient | | | 🗶 | | The term ‘wear and tear’ may not be a proxy for the belief that exercise will damage the joint further | |  |
| Risk factors for CKP | | | 🗶 | | Trend towards increased exercise use if believe that risk factors modifiable and decreased exercise use if believe risk factors to be unmodifiable | |  |
| Beliefs about the future for patients with CKP | | | 🗶 | |  | |  |
| Used knee x-ray for the vignette patient | | | 🗶 | |  | |  |
| Agreement that exercise is effective if the knee x-ray shows severe knee OA | | | 1.97 (1.24, 3.15)* | | Disagreement is not significantly associated* | |  |
| Agreement that exercise works just as well for everybody, regardless of the amount of pain they have | | | 🗶 | | Disagreement is not significantly associated* | |  |
| **Knowledge about the risks/safety of exercises** | | | | | | |  |
| Agreement that quadriceps strengthening exercises for the knee are safe for everybody to do | | | 2.01 (1.29, 3.15)* | | Disagreement is not significantly associated* | |  |
| Agreement that general exercise, for example walking or swimming is safe for everybody to do | | | 1.99 (1.21, 3.28)* | | Disagreement is not significantly associated* | |  |
| Prior experience of being uncertain about the safety of exercise as a barrier to using exercise | | | 🗶 | | Small numbers, trend towards reduced exercise use if this barrier experienced | |  |
| Biomedical treatment orientation subscale score in top 25% | | | 🗶 | | Trend towards lower use of exercise among those with top 25% scores^ | |  |
| Behavioural treatment orientation subscale score in top 25% | | | 1.87 (1.03, 3.39)^ | | Indication that scores on this subscale may differentiate GPs whose attitudes are more in line with evidence-based exercise recommendations | |  |
| **BELIEFS ABOUT MORAL NORM** | | | | | | |  |
| GPs should prescribe quadriceps strengthening exercises to every patient with CKP | | | | 3.08 (1.96, 4.83)* | | Disagreement not significantly associated* | |
| GPs should prescribe general exercise, for example, walking or swimming, for every patient with CKP | | | | 2.63 (1.45, 4.76)* | | Disagreement not significantly associated* | |
| **BELIEFS ABOUT CAPABILITIES** | | | | | | |  |
| **GP-related factors** | | | | | | | |
| Prior experience of being uncertain about the most appropriate type of exercise to use as a barrier to using exercise | | 0.38 (0.25, 0.58) | | | |  | |
| Prior experience of having insufficient expertise to give detailed information as a barrier to using exercise | | 0.50 (0.33, 0.76) | | | |  | |
| Agreement that exercise for CKP is more effectively provided by physiotherapists than GPs | | 🗶 | | | | Trend towards increased use of exercise among those who disagree and decreased use among those who agree | |
| **Service-related factors** | | | | | | | |
| Agreement that GPs have enough time to manage patients with CKP | | 🗶 | | | | Disagreement not significantly associated* | |
| Agreement that time constraints prevent GPs from providing advice on individual exercises for CKP | | 🗶 | | | | Disagreement not significantly associated* | |
| Agreement that exercise for CKP would be used more frequently if access to physiotherapy was easier | | 🗶 | | | | Disagreement not significantly associated* | |
| Prior experience that there is insufficient time in consultations as a barrier to using exercise | | 🗶 | | | |  | |
| Prior experience of difficulty accessing physiotherapy as a barrier to using exercise | | 🗶 | | | |  | |
| **Patient-related factors** | | | | | | | |
| Prior experience that patients prefer other management options as a barrier to using exercise | | 🗶 | | | |  | |
| Prior experience that exercise does not match patient needs and/or expectations | | 🗶 | | | |  | |
| **CHARACTERISTICS OF THE GPS** | | | | | | | |
| Gender | | Male  0.64 (0.42, 0.97) | | | | Compared with females | |
| Time since qualification | | 🗶 | | | |  | |
| Type of GP | | 🗶 | | | |  | |
| Number of GPs in practice | | 🗶 | | | |  | |
| Practice type | | 🗶 | | | |  | |
| GPwSI | | Pearson Chi-squared = 7.694, df 1, p=0.006 | | | | OR could not be calculated | |
| Postgraduate MSK training | | 🗶 | | | |  | |
| Personal experience of CKP | | 🗶 | | | |  | |
| **BELIEFS ABOUT SOCIAL INFLUENCES** | | | | | | |  |
| Experience of GP colleagues not using or valuing exercise as a barrier to using exercise | | | | 🗶 | | Small numbers | |
| **BEHAVIOURAL INTENTION** | | | | | | | |
| **Motivation and goals** | | | | | | | |
| Agreeing that managing patients with CKP is of clinical interest to me | 🗶 | | | | | Trend towards increased use among those agreeing it is of interest | |
| Agreeing that managing patients with CKP is a priority to me | 🗶 | | | | | Trend towards increased use among those agreeing it is a priority and decreased use among those who disagree | |
| *Compared with use of exercise among those responding with neither disagree or agree; **Compared with severe/very severe; ^Compared with those with scores in bottom 25%. CKP = chronic knee pain; GP = general practitioner; GPwSI = general practitioner with special interest; MSK = musculoskeletal; OA = osteoarthritis; OR = odds ratio | | | | | | |  |
