# Supplementary material for: What influences general practitioners’ use of exercise for patients with chronic knee pain? Results from a national survey
Source: BMC Fam Pract. 2016 Dec 19;17:172. doi: 10.1186/s12875-016-0570-4 (PMC5168590; doi:10.1186/s12875-016-0570-4)
Supplement: Additional file 4: — Association between perception of time constraints and delivery method for exercises. (DOCX 16 kb) [file 12875_2016_570_MOESM4_ESM.docx]

Additional File 4 Association between perception of time constraints and delivery method for exercises

Table AF 4-1 Association between perception of time constraints and delivery method for general exercises

| Time constraints prevent GPs from providing advice on individual exercises for CKP | General exercise initiation method | | | | | | | |  |
| --- | --- | --- | --- | --- | --- | --- | --- | --- | --- |
|  | **Suggests** | | **OR (95% CI)** | **Provides leaflet** | | **OR (95% CI)** | **Refers** | | **OR (95% CI)** |
|  | **No** | **Yes** |  | **No** | **Yes** |  | **No** | **Yes** |  |
| Neither disagree nor agree | 30 (48%) | 33 (52%) | 1.00 | 27 (44%) | 35 (57%) | 1.00 | 46 (73%) | 17 (27%) | 1.00 |
| (Strongly) disagree | 18 (30%) | 43 (71%) | **2.17 (1.04,4.55)** | 36 (59%) | 25 (41%) | 0.54 (0.26,1.10) | 50 (82%) | 11 (18%) | 0.60 (0.25,1.40) |
| (Strongly) agree | 241 (45%) | 290 (55%) | 1.09 (0.65,1.85) | 265 (50%) | 265 (50%) | 0.77 (0.45,1.31) | 359 (68%) | 171 (32%) | 1.29 (0.72,2.31) |

Table AF 4-2 Association between perception of time constraints and delivery method for local exercises

| Time constraints prevent GPs from providing advice on individual exercises for CKP | Local exercise initiation method | | | | | | | |  |
| --- | --- | --- | --- | --- | --- | --- | --- | --- | --- |
|  | **Demonstrates** | | **OR (95% CI)** | **Provides leaflet** | | **OR (95% CI)** | **Refers** | | **OR (95% CI)** |
|  | **No** | **Yes** |  | **No** | **Yes** |  | **No** | **Yes** |  |
| Neither disagree nor agree | 30 (47%) | 34 (53%) | 1.00 | 28 (44%) | 36 (56%) | 1.00 | 46 (72%) | 18 (28%) | 1.00 |
| (Strongly) disagree | 20 (29%) | 49 (71%) | **2.16 (1.06,4.42)** | 38 (55%) | 31 (45%) | 0.64 (0.32,1.26) | 57 (83%) | 12 (17%) | 0.54 (0.24,1.23) |
| (Strongly) agree | 233 (47%) | 259 (53%) | 0.98 (0.58,1.65) | 216 (44%) | 276 (56%) | 0.99 (0.59,1.68) | 335 (68%) | 157 (32%) | 1.20 (0.67,2.13) |
